# Eye hemodynamic data and biochemical parameters of the lacrimal fluid of patients with non-proliferative diabetic retinopathy

**DOI:** 10.1016/j.dib.2020.106237

**Published:** 2020-08-27

**Authors:** Guzal Kangilbaeva, Fazilat Bakhritdinova, Iroda Nabieva, Aziza Jurabekova

**Affiliations:** a Department of Ophthalmology, Tashkent Medical Academy, Tashkent, Uzbekistan; b Clinic of Research Institute of Endocrinology, Tashkent, Uzbekistan; c Nazar Medical Eye Clinic, Tashkent, Uzbekistan

**Keywords:** Diabetic retinopathy, Antioxidant system in the tear, Doppler ultrasound imaging, Correlation coefficients

## Abstract

This paper presents a data from examinations of patients treated in clinic of Tashkent medical academy, Uzbekistan. 165 Patients (305 eyes) with NPDR were randomly allocated to receive standard treatment as the control group, in addition to the standard treatment daily tablets of EGb 761 (Tanakan, Beaufour Ipsen Industrie, Paris, France) 120 mg as the 1st main group, or daily endonasal electrophoresis of Tanakan 40 mg as 2nd main group, within 10 days. All of the patients underwent baseline ophthalmologic examinations, definition of biochemical parameters of the lacrimal fluid and Doppler ultrasound imaging of the eye. Moreover, correlation between tear fluid and hemodynamics was calculated. This data is essential for researchers to develop diagnostic criteria for diabetic retinopathy stages. Data are also beneficial to practitioners in determining the diabetic retinopathy severity and choosing appropriate treatment.

**Specifications Table**

**Table:** 

Subject	Ophthalmology
Specific subject area	Diabetic retinopathy, human retina, Biochemical parameters of the lacrimal fluid, antioxidant system in the tear, Doppler ultrasound imaging of the eye, hemodynamic parameters, correlation coefficients
Type of data	Table
How data were acquired	slit-lamp fundus examinations using 60, 90D (Volk) lenses, Goldman three-mirror lens, and Mainster Standart fundus lens, ultrasound system HD 11XE (Philips) and HI VISION Preirus (Hitachi). mass spectrometry, spectrofotometr SF-46
Data format	Raw Analyzed Filtered
Parameters for data collection	hard exudates, cotton wool spots, retinal hemorrhages, microaneurysms, venous anomalies, and macular edema
Description of data collection	Fundus images, Mean peak systolic velocity (PSV) in ocular vessels, antioxidant system of the lacrimal fluid
Data source location	The data under investigation was obtained from Tashkent Medical Academy, Tashkent, Uzbekistan. 41.350526°N, 69.174250°E
Data accessibility	Repository name: Features of clinical and functional indicators of the eye with diabetic retinopathy Data identification number:http://dx.doi.org/10.17632/d56n7mmr2t.3 Direct URL to data:https://data.mendeley.com/datasets/d56n7mmr2t/3

**Value of the Data**

• These data show changes in the biochemical composition of the tears of patients with non-proliferative diabetic retinopathy.
• These data show, that blood flow velocity changes with increasing severity of diabetic retinopathy.
• These data can help ophthalmologists to make the correct diagnosis and stage of the disease.
• Correlation coefficients between diabetic retinopathy severity, the level of antioxidant protection system in the tear, and blood flow velocity valuable for the development of disease prognosis.

## 1 Data Description

Table 1 shows, that mean diabetic retinopathy severity (DRS) reduction in the 2nd main group was statistically significant compared with the baseline value (*p* <0.01) after treatment and remained at low values for 6 months [1]. On the contrary, in the control group, the decrease in DR severity after treatment was not statistically significant (*p*> 0.05), and an increase in the severity of DR was observed at month 6.

**Table 1:** Change in mean diabetic retinopathy severity during observation. (Mean±SD).

NPDR Stage	Groups	Baseline	Day 10	Month 1	Month 3	Month 6
NPDR Ia	Control (*n*=32)	26,03±5,5	22,5±10,2	22,5±9,3	26,5±11,6	30,5±9,5*
	1st main (*n*=31)	27,3±5,5	20,2±7,1⁎⁎	19,4±7,9⁎⁎	20,6±7,5⁎⁎^,^#	23,2±8,6*^,^#
	2nd main (*n*=33)	27,1±6,3	21,8±8,1⁎⁎	20,9±9,3⁎⁎	21,4±9,5⁎⁎^,^#	21,5±9,6⁎⁎^,^#
NPDR Ib	Control (*n*=46)	42.9±10.9	38.2±10.4*	38,8±10,6	43,9±11,7	52,7±15,3*
	1st main (*n*=46)	40,2±8,3	32,8±8,7⁎⁎^,^#	31±10,1⁎⁎^,^#	32,6±8,3⁎⁎^,^#	34,9±8,1⁎⁎^,^#
	2nd main (*n*=44)	41,6±9,4	30,9±7,6⁎⁎^,^#	29,5±8,5⁎⁎^,^#	29,2±9,1⁎⁎^,^#	30,9±8,94⁎⁎^,^#^,^^
NPDR Ic	Control (*n*=23)	67,04±7,2	64,5±10,6	64,5±8,8	70,04±10,5	83,5±5,8⁎⁎
	1st main (n=23)	62±13,7	54,8±15,0#	52,4±17,9*^,^#	55,7±17,6#	60,4±22,0#
	2nd main (*n*=28)	66,4±17,7	49,3±14,4⁎⁎^,^#	46,6±12,1⁎⁎^,^#	46,1±12,3⁎⁎^,^#^,^^	45,7±12,8⁎⁎^,^#^,^^

NPDR= non-proliferative diabetic retinopathy.

⁎ *р* < 0.05.

⁎⁎ *р* < 0.01 – significant differences from baseline.

# *р* < 0.05 – significant differences from control group.

^ *р* < 0.05 – significant differences from 1st main group; SD= Standard deviation.

The antioxidant system in the tear of eye with DR is changed [2,3]. Our studies have shown a gradual decrease in NO levels as DRS increases. Table 2 show statistically significant (*р* < 0.05) NO reduction and MDA increases from healthy group.

**Table 2:** Biochemical parameters of the lacrimal fluid of patients with NDR by stages. (Mean±SD).

parameters	Healthy *n*=10	NPDR stages			Correlation with stage.
		1–a, *n*=27	1–b, *n*=31	1–c, *n*=12	
NO, nmol/ml	3,54±0,8	2,82±0,85*	1,86±0,62*^,^#	1,17±0,42*^,^#^,^^	*r*= −0,65 *p*=0,0001
MDA, nmol/ml	1,48±0,2	3,86±0,44*	4,13±0,41*^,^#	4,64±1,34*^,^#	*r*= 0,37 *p*=0,07
catalase activity, MU/I	0,19±0,04	0,11±0,06*	0,09±0,05*	0,08±0,03*^,^#	*r*= −0,22 *p*=0,27
superoxide dismutase (SOD) activity, nmol/min/ml	6,8±0,6	3,71±0,68*	3,63±0,76*	3,08±0,39*^,^#^,^^	*r*= −0,23 *p*=0,25

NO= Nitric oxide; MDA=malondialdehyde.

⁎ *Р*<0.05 – from healthy.

# *Р*<0.05 – from NPDR Ia.

^ *Р*<0.05 – from NPDR Ib (*Р*<0.05). SD= Standard deviation.

Table 3 shows progressive decrease in blood flow in the second Ib and third Ic stage of NPDR in the central retinal artery (CRA) at baseline [4,5]. Also, a progressive decrease in blood flow by stages was detected in the short posterior ciliary artery (SPCA). Peak systolic velocity (PSV) reduction in Ib and Ic stage was statistically significant (Р<0.05) from healphy.

**Table 3:** Baseline hemodynamic parameters of eyes with NDR (Mean±SD).

	CRA PSV	CRA RI	CRV PSV	SPCA PSV	SPCA RI	OA PSV	OA RI
Healthy	13,7±0,3	0,68±0,01	7,48±1,02	14,8±0,3	0,67±0,01	41,7±0,7	0,76±0,01
NPDR Ia	14,8±5,64	0,72±0,05⁎⁎	6,48±1,53	11,4±6,38*	0,66±0,04	30,1±16,5⁎⁎	0,73±0,13
NPDR Ib	7,3±1,54⁎⁎^,^#	0,73±0,04⁎⁎	4,58±1,94*^,^#	10,6±2,4⁎⁎	0,71±0,03⁎⁎^,^#	44,4±12,3#	0,73±0,09
NPDR Ic	5,1±1,18⁎⁎^,^#^,^^	0,66±0,05#^,^^	3,97±1,64⁎⁎^,^#	10,2±2,1⁎⁎	0,72±0,05⁎⁎^,^#	36,7±8,1*^,^^	0,77±0,08

NPDR=nonproliferative diabetic retinopathy, PSV =peak systolic blood flow velocity, CRA=central retinal artery, CRV=central retinal vena, SPCA=short posterior ciliary artery, OA=ophthalmic artery, RI =resistive index.

⁎ *р* < 0.05.

⁎⁎ *р* < 0.01 – from healthy.

# *Р*<0.05 – from NPDR Ia, ^*Р*<0.05– from NPDR Ib; SD= Standard deviation.

In the correlation analysis (Table 4), a strong positive correlation was found (*r* = 0.71) between PSV in the CRA and the level of NO in the eyes with the NPDR Ia stage. Moderate positive correlation was found between the PSV of the CRA and the level of NO in the eyes of NDR Ib and NPDR Ic, as well as between EDV in the CRA and the level of NO in the eyes of NPDR Ia (*r* = 0.67). In addition, moderate positive correlations were found between RI CRA and catalase activity in NDR Ia *(r* = 0.45), SOD activity in NDR Ib (*r* = 0.46), between PSV in CRV and the level of NO (*r* = 0.44 and *r* = 0.32) in the NPDR Ia and the NDR Ib stages. Moderate negative correlations were found between the level of MDA and PSV in the CRA (*r* = −0.44, *r* = −0.56), CRV (*r* = −0.34), the EDV in the SPCA (*r* = −0.41) and OA (*r* = −0.31).

**Table 4:** Pearson's correlation coefficients between hemodynamic parameters and biochemical parameters of the lacrimal fluid.

		CRA				CRV	SPCA				OA				ORC
		PSV	EDV	RI	PI	CRA	CRA	EDV	RI	PI	CRA	EDV	RI	PI	
NPDR Ia	NO	0.71	0.67	−0.09	−0.29	0.44	0.11	0.15	−0.13	−0.17	−0.02	−0.19	0.10	0.08	0.11
	MDA	−0.44	−0.41	0.02	0.13	−0.22	−0.22	−0.28	0.24	0.29	−0.05	0.21	−0.24	−0.27	−0.23
	Catalase	−0.24	−0.27	0.45	0.04	−0.13	0.23	0.19	0.20	0.35	0.25	0.12	0.20	−0.12	0.02
	SOD	0.32	0.33	0.06	−0.13	0.02	0.04	0.05	−0.09	−0.12	−0.02	−0.17	0.10	−0.10	0.07
NPDR Ib	NO	0.35	0.33	0.01	0.38	0.32	−0.07	0.01	−0.14	−0.24	−0.23	−0.42	0.52	0.44	0.49
	MDA	0.16	0.26	−0.15	0.16	−0.01	0.10	0.03	0.17	−0.23	0.19	0.02	0.12	0.18	0.18
	Catalase	0.11	0.01	0.10	−0.02	0.14	−0.21	−0.15	−0.09	0.10	−0.07	−0.09	0.08	0.21	0.03
	SOD	0.21	−0.18	0.46	0.32	0.04	−0.14	−0.29	0.27	0.52	0.06	0.20	−0.33	−0.13	−0.49
NPDR Ic	NO	0.43	0.34	0.10	0.34	−0.24	−0.36	−0.30	0.17	0.15	0.25	0.13	−0.03	0.37	−0.07
	MDA	−0.56	−0.25	−0.30	−0.26	−0.34	−0.27	−0.41	0.47	0.31	−0.21	−0.31	0.33	0.38	0.48
	Catalase	−0.20	−0.09	0.06	−0.06	−0.03	0.07	0.05	−0.03	0.05	0.08	0.19	−0.20	−0.21	−0.21
	SOD	−0.29	−0.22	−0.15	−0.21	−0.27	−0.21	−0.25	0.27	0.27	0.01	0.07	−0.08	0.05	0.03

NPDR=nonproliferative diabetic retinopathy, PSV =peak systolic blood flow velocity, EDV=end-diastolic velocity; CRA=central retinal artery, CRV=central retinal vena, SPCA=short posterior ciliary artery, OA=ophthalmic artery, RI =resistive index; PI=pulsatility index; NO= Nitric oxide; MDA=malondialdehyde; SOD= superoxide dismutase.

Strong and moderate correlations coefficients are marked as underlined text in the table.

## 2 Experimental design, materials and methods

This data is collected from examinations of 165 patients (305 eyes) in the clinic of Tashkent medical academy. Patients had non-proliferative diabetic retinopathy (NPDR). Age ranged from 18 to 79 years, women were 94, men 71. The exclusion criteria were eye diseases such as glaucoma, inflammatory eye diseases, pigmented and other retinal dystrophies, retinal detachments, a period not earlier than 6 months after an eye injury or eye surgery, as well as severe somatic diseases such as kidney or liver failure, glucose-galactose malabsorption (GGM), hypertensive crisis.

Patients with NPDR were randomly allocated to receive standard treatment (hypoglycemic treatment, nootropil, statins, fenofibrats, angioprotectors) as the control group, in addition to the standard treatment daily tablets of EGb 761 (Tanakan, Beaufour Ipsen Industrie, Paris, France) 120 mg as the 1st main group, or daily endonasal electrophoresis of Tanakan 40 mg as the 2nd main group, within 10 days. The patients underwent anterior segment slit-lamp and fundus examinations using 60, 90D (Volk) lenses, Goldman three-mirror lens, and Mainster Standart fundus lens, the antioxidant activity of tears examinations, and Doppler ultrasound imaging of the eye.

The stages of DR were determined according to the severity scale of the Early Treatment Diabetic Retinopathy Study (ETDRS), the "gold" standard for a detailed assessment of the fundus condition in scientific studies worldwide. All statistical analyses were performed using the Statistical Package for the Social Sciences (SPSS) software version 23. P values < 0.05 were considered statistically significant.

## Ethics statement

Permission of the National Ethics Committee of the Ministry of Health of the Republic of Uzbekistan No. 11 of 2.11.2010

All patients signed informed consent for treatment and examination

## Declaration of Competing Interest

The authors declare that they have no known competing financial interests or personal relationships that could have appeared to influence the work reported in this paper.

## Appendix Supplementary materials

Image, application 1

Image, application 2
